# Characterization of the functional effects of ferredoxin 1 as a cuproptosis biomarker in cancer

**DOI:** 10.3389/fgene.2022.969856

**Published:** 2022-09-26

**Authors:** Xiang Li, Zihan Dai, Jincheng Liu, Zhenqian Sun, Na Li, Guangjun Jiao, Hongxin Cao

**Affiliations:** ^1^ Cheeloo College of Medicine, Shandong University, Jinan, China; ^2^ Mechanics Laboratory, Binzhou Medical University, Yantai, China; ^3^ Department of Orthopedics, Qilu Hospital, Shandong University, Jinan, China; ^4^ Department of Medical Oncology, Qilu Hospital, Shandong University, Jinan, China

**Keywords:** tumor, cuproptosis, ferredoxin 1, pan-cancer analysis, biomarker

## Abstract

**Background:** Cuproptosis is a recently discovered form of programmed cell death. Ferredoxin 1 (FDX1) is a key gene that mediates this process. However, the role of FDX1 in human tumors is not clear.

**Methods:** We comprehensively analyzed the differential expression and genetic alterations of FDX1 using multiomics data from The Cancer Genome Atlas (TCGA) and the Genotype-Tissue Expression (GTEx) database. Subsequently, we explored the association between FDX1 and tumor parameters such as genomic instability, RNA methylation modifications, immune infiltration and pathway activity. In addition, we performed functional enrichment analysis and assessed the sensitivity potential of FDX1-related drugs. Finally, we experimentally verified the functional effects of FDX1.

**Results:** The analysis revealed differential expression of FDX1 in a variety of tumors. By analyzing the association of FDX1 expression with genomic instability, immune cell infiltration, signaling pathway *etc.* We explored the role of FDX1 in regulating cell activity. Also, we evaluated the function of FDX1 in biologic process and drug sensitivity. Our experimental results demonstrated that FDX1 exerts its antitumor effects through cuproptosis in liver hepatocellular carcinoma and non-small cell lung cancer cell lines.

**Conclusion:** Our study reveals the functional effects of FDX1 in tumors and deepens the understanding of the effects of FDX1. We validated the inhibitory effect of FDX1 in copper induced cell-death, confirming the role of FDX1 as a cuproptosis biomarker.

## Introduction

Cancer is one of the most important public health problems in the world and has become a major impediment to increasing human life expectancy (Siegel et al., 2022). However, the prognosis of many tumors remains poor and patient survival rates are low due to factors such as drug resistance. Some of the main mechanisms leading to the emergence of this drug resistance are tumor evasion and resistance to programmed cell death (PCD) (Johnstone et al., 2002). PCD includes necroptosis, pyroptosis, ferroptosis, and cuproptosis (Bertheloot et al., 2021). As a recently discovered mode of cell death, cuproptosis undoubtedly has great potential in the treatment of tumors.

Unlike other types of PCD, ferredoxin 1 (FDX1) and protein lipoylation are the key regulators of copper ionophore induced cell death (Tang et al., 2022). Copper is an essential nutrient, but it is both beneficial and toxic to cells. The link between copper and disease has been observed in previous studies, and some studies have reported that dysregulation of copper homeostasis played an important role in tumors (Oliveri, 2022). However, it is not certain whether copper is a cause or a consequence of tumorigenesis (Ge et al., 2022). An in-depth analysis of the mechanism of cuproptosis was carried out in the pioneering work of Tsvetkov *et al.* FDX1 was a direct target of copper ion carriers, and the researchers found that knockdown of FDX1 could protect cells from copper toxicity (Tsvetkov et al., 2022). In terms of biological function, FDX1 is a protein-coding gene. It is deeply involved in mitochondrial respiration and steroid metabolism (Sheftel et al., 2010; Strushkevich et al., 2011). And mitochondrial respiration regulates copper ionophore induced cell death. Thus, FDX1 is the main molecule that mediates cuproptosis (Tsvetkov et al., 2022). These work reveals the biological significance of copper and inspires a new strategy for tumor treatment: targeting cuproptosis. And FDX1 is the key gene related to such strategies.

The role of FDX1 in human diseases including tumors is not well known, and the effect of FDX1 mediated cuproptosis in tumors is not clear (Zhang et al., 2021). Therefore, a large-scale pan-cancer analysis of FDX1 is necessary to explore the therapeutic potential of strategies targeting cuproptosis for tumor treatment. In this study, we investigated FDX1 expression and its association with RNA methylation modifications, genomic instability, immune status, pathway activity and prognosis. Furthermore, we predicted immunotherapeutic responses and effective small molecule drugs based on the expression of FDX1 with public datasets. Finally, a series of molecular experiments such as CCK8, ethynyldeoxyuridine (EdU) staining and colony formation assay were performed to validate the role of FDX1 and cuproptosis in tumors.

## Methods and materials

### Expression alteration and survival analysis

We used ProteomicsDB to investigate the gene and protein expression of FDX1 in normal tissues (Schmidt et al., 2018). Clinical features and mRNA expression profiles of 33 human tumors were downloaded from TCGA (Weinstein et al., 2013). The mRNA expression matrix of normal tissues was obtained from GTEx (GTEx Consortium, 2017). UALCAN was used to assess the protein expression level of FDX1 (Chandrashekar et al., 2022).

Cox regression analysis was used to analyze the relationship between FDX1 expression and patient survival. Parameters for the survival analysis included overall survival (OS), progression-free survival (PFS), disease -specific survival (DSS) and disease-free interval (DFI).

### Genetic alteration and RNA methylation modification analyses

We performed genetic alteration analysis with cBioPortal and GSCA (Cerami et al., 2012; Liu et al., 2018). Genetic alteration parameters included FDX1 mutation type, spectrum and frequency in tumors, and the relationship between mutations and patient survival was analyzed. In addition, the copy number variations (CNVs), single nucleotide variations (SNVs) and methylation level of FDX1 were analyzed.

RNA methylation modifications mainly include N1-methyladenosine (m1A), 5-methylcytidine (m5C) and N6-methyladenosine (m6A). Modification-related genes are classified as writers, readers and erasers. We explored the relationship between FDX1 expression and genes related to RNA methylation modifications in different tumor types.

### Genomic instability and immune infiltration analyses

Here, genomic instability was assessed based on tumor mutation burden (TMB), microsatellite instability (MSI), DNA stemness score (DNAss), RNA stemness score (RNAss), homologous recombination deficiency (HRD) status, loss of heterozygosity (LOH) status, mutant-allele tumor heterogeneity (MATH) score and neoantigen (NEO) levels. Sangerbox, an online data analysis platform, was used to assess the relationship of FDX1 expression with genomic instability in tumors.

For immune infiltration analysis, expression data of common immune checkpoints were extracted from TCGA. CIBERSORT, MCPcounter and TIMER were used to assess the levels of immune cell infiltration in TCGA. The correlations between the levels of infiltrating immune cells and FDX1 expression were calculated using Spearman correlation analysis.

### Evaluation of the effect of FDX1 on biological processes in tumors

We evaluated the effect of FDX1 on pathway activity based on previous studies (Akbani et al., 2014; Ye et al., 2018). The pathways involved in this analysis included well-known cancer-related pathways such as the apoptosis, cell cycle, and DNA damage pathways. The pathway score was defined as the sum of the relative protein levels of all positively regulated genes minus the sum of the relative protein levels of all negatively regulated genes. A score greater than zero indicated that FDX1 activated the pathway, and a score less than zero indicated that FDX1 inhibited the pathway.

The tumor microenvironment (TME) scores were calculated by the ESTIMATE method using TCGA data (Yoshihara et al., 2013). In the TCGA cohort, the effect of FDX1 on tumor proliferation was assessed by analyzing the correlation between FDX1 expression and the Ki67 level.

### FDX1-associated gene enrichment analysis

We used STRING to obtain genes associated with FDX1 and constructed a protein–protein interaction network (Szklarczyk et al., 2021). The parameters were set to the default values. GEPIA2 was used to obtain the top 30 genes associated with FDX1 in tumors (Tang et al., 2017). A gene co-expression network was generated by GeneMANIA (Warde-Farley et al., 2010). In addition, we obtained genes associated with cuproptosis from Tsvetkov’s work (Tsvetkov et al., 2022). We pooled the genes obtained from the four approaches and removed the duplicates. Then, Gene Ontology (GO) and Kyoto Encyclopedia of Genes and Genomes (KEGG) enrichment analyses of these genes were performed. The GO analysis included analysis of biological process (BP), cellular component (CC) and molecular function (MF) terms. Finally, the OPENTARGET platform was used to identify the relationship between FDX1 and diseases.

### Drug sensitivity analysis

GSCA was used to analyze the relationship between drug sensitivity and gene expression using data from cancer cell lines in GDSC and CTRP (Yang et al., 2013; Rees et al., 2016; Liu et al., 2018). Pearson correlation analysis was used to assess the relationship between the expression of FDX1 in and small molecule drug sensitivity (IC50 values). In addition, the Tumor Immune Dysfunction and Exclusion (TIDE) algorithm was applied to assess the association of FDX1 expression with immunotherapy response (Fu et al., 2020).

### Cell culture and transfection

Two human tumor cell lines were used in this study: HepG2 (liver hepatocellular carcinoma) and H1299 (non-small cell lung cancer). The cell lines were purchased from Procell Biotechnology Co., Ltd. (Wuhan, China) and cultured according to the instructions. The identity of the cell lines was confirmed by short tandem repeat (STR) analysis.

Small interfering RNAs were purchased from Gene Pharma (Shanghai, China). The following siRNA sequences were used: FDX1-1-F: GUG​AUU​CUC​UGC​UAG​AUG​UTT; FDX1-1-R: ACA​UCU​AGC​AGA​GAA​UCA​CTT; FDX1-2-F: CCU​GUC​ACC​UCA​UCU​UUG​ATT; FDX1-2-R: UCA​AAG​AUG​AGG​UGA​CAG​GTT; FDX1-3-F: CUA​ACA​GAC​AGA​UCA​CGG​UTT; FDX1-3-R: ACC​GUG​AUC​UGU​CUG​UUA​GTT.

We transfected the cells according to the manufacturer’s protocol. After that, PCR was used to confirm the transfection effect. β-Actin was set as internal control and relative gene expression was calculated by the 2^–ΔΔCt^ method. The primers were as follows: FDX1-F, TTC​AAC​CTG​TCA​CCT​CAT​CTT​TG; FDX1-R, TGC​CAG​ATC​GAG​CAT​GTC​ATT.

### Analysis of cell proliferation

After 48 h of transfection, we added CuSO_4_ (1 μM), elesclomol (ELE) (100 nM) and tetrathiomolybdate (TTM) (100 nM) to the culture medium to trigger FDX1-mediated cuproptosis. ELE is a copper ion carrier, while TTM is a copper ion chelator. Cell proliferation was detected by CCK-8, ethynyldeoxyuridine (EdU) staining and colony formation assay.

For CCK-8, the cell lines and controls were first inoculated separately in 96-well plates. 10 μL of CCK-8 reagent was added to each well at the specified time points. The optical density (OD) was measured at 450 nm after incubation for 1 h at 37°C.

The fraction of DNA-replicating cells, which represents cell proliferation status, was assessed using EdU detection kit (RiboBio, Guangzhou, China).

For colony formation assay, cells were incubated in a 6-well plate at 37°C for 1 week, followed by fixation in methanol and staining in a 0.1% crystal violet solution for 15 min before colony counting.

### Statistics analysis

In bioinformatics analysis, we used flexible methods to process data. For molecular biology experiments, two-sided student’s t-test was used to compare the difference between two groups. Mean ± standard deviation was used to represent quantitative data. In correlation tests, the absolute value of the correlation coefficient ≥0.7 would be regarded as “strong” correlation, values between 0.50 and 0.70 would be interpreted as “good” correlation, between 0.3 and 0.5 would be treated as “fair” or “moderate” correlation, and any value ≤0.30 would be poor correlation (Hazra and Gogtay, 2016). Difference with *p* < 0.05 was considered statistically significant.

## Results

### Dysregulation and prognostic value of FDX1 in tumors

**FIGURE 1:**
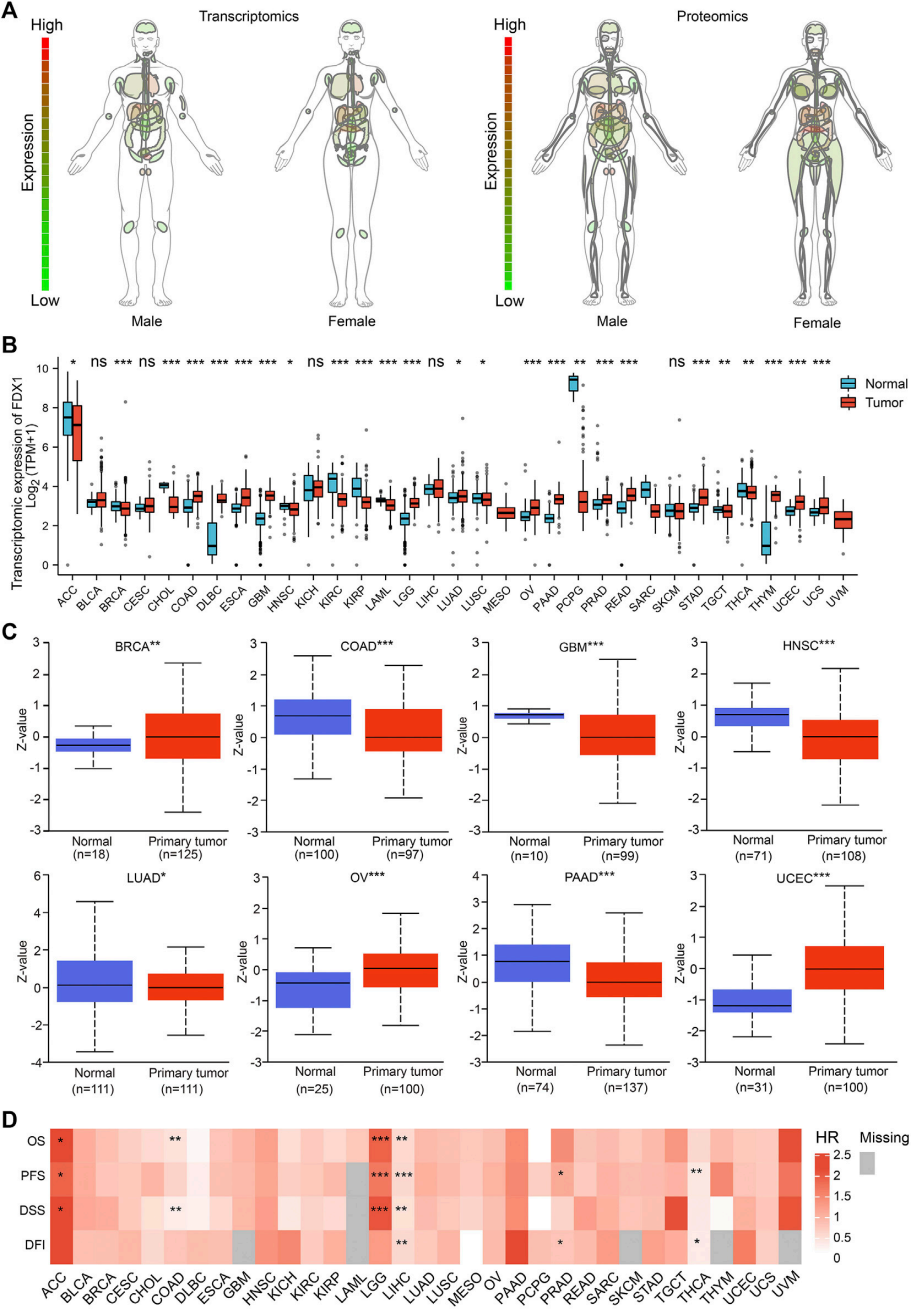
FDX1 expression profile and survival analysis. **(A)** Anatomy plot of the transcriptomic and proteomic expression of FDX1 across normal tissues in males and females. **(B)** Combining the data from TCGA and GTEx, transcriptomic expression differences of FDX1 between tumor and normal tissues. (ns: not significant; *p < 0.05; **p < 0.01; ***p < 0.001). **(C)** Data from UALCAN revealed proteomic expression differences of FDX1 between tumor and normal tissues. (*p < 0.05; **p < 0.01; ***p < 0.001). **(D)** Survival analysis across tumors revealed FDX1 was associated with prognosis. (HR, hazard ratio; OS, overall survival; PFS, progression free survival; DSS, disease specific survival; DFI, disease free interval; *p < 0.05; **p < 0.01; ***p < 0.001).

First, analysis of normal tissues showed that the gene and protein expression of FDX1 varies in different organs and tissues, with the highest expression level in adrenal gland in both males and females (Figure 1A). As shown in Figure 1B, the RNA expression of FDX1 differed significantly in 25 of the 33 tumor types studied. Analysis of proteomic data also revealed differential expression of FDX1 in the tumors, among which FDX1 expression level was lower in colon adenocarcinoma (COAD), glioblastoma multiforme (GBM), head and neck squamous cell carcinoma (HNSC), lung adenocarcinoma (LUAD) and pancreatic adenocarcinoma (PAAD) compared with normal tissues; while higher in breast invasive carcinoma (BRCA), ovarian serous (OV) and uterine corpus endometrial carcinoma (UCEC) (Figure 1C).

As shown in Figure 1D, Cox regression analysis showed that the expression of FDX1 was positively correlated with the prognosis of adrenocortical carcinoma (ACC) and lower grade glioma (LGG); and negatively correlated with the prognosis of colon adenocarcinoma (COAD), liver hepatocellular carcinoma (LIHC) and thyroid carcinoma (THCA). The abbreviations and full names of all the tumors mentioned in this study are presented in Table 1. Taken together, our data indicated that the expression of FDX1 was dysregulated in tumors and that this dysregulation had prognostic implications in some tumors.

**TABLE 1:** Abbreviations and full names of cancer types used in this study.

Abbreviation	Full name	Abbreviation	Full name
ACC	Adrenocortical carcinoma	MNT	Miscellaneous neuroepithelial tumor
BLCA	Bladder urothelial carcinoma	NSCLC	Non-small cell lung cancer
BRCA	Breast invasive carcinoma	NSGCT	Non-seminomatous germ cell tumor
CEAD	Cervical adenocarcinoma	OET	Ovarian epithelial tumor
CESC	Cervical squamous cell carcinoma and endocervical adenocarcinoma	OM	Ocular melanoma
CHOL	Cholangiocarcinoma	OV	Ovarian serous cystadenocarcinoma
COAD	Colon adenocarcinoma	PAAD	Pancreatic adenocarcinoma
COADREAD	Colon adenocarcinoma/Rectum adenocarcinoma Esophageal carcinoma	PCPG	Pheochromocytoma and paraganglioma
DG	Diffuse glioma	PRAD	Prostate adenocarcinoma
DLBC	Lymphoid neoplasm diffuse large B-cell lymphoma	READ	Rectum adenocarcinoma
EA	Esophagogastric Adenocarcinoma	RNCCC	Renal non-clear cell carcinoma
ESCA	Esophageal carcinoma	SARC	Sarcoma
GBM	Glioblastoma multiforme	SEMI	Seminoma
HNSC	Head and neck squamous cell carcinoma	SKCM	Skin cutaneous melanoma
KICH	Kidney chromophobe	STAD	Stomach adenocarcinoma
KIRC	Kidney renal clear cell carcinoma	STES	Stomach and esophageal carcinoma
KIRP	Kidney renal papillary cell carcinoma	TET	Thymic epithelial tumor
LAML	Acute myeloid leukemia	TGCT	Testicular germ cell tumors
LGG	Brain lower grade glioma	THCA	Thyroid carcinoma
LIHC	Liver hepatocellular carcinoma	THYM	Thymoma
LUAD	Lung adenocarcinoma	UCEC	Uterine corpus endometrial carcinoma
LUSC	Lung squamous cell carcinoma	UCS	Uterine carcinosarcoma
MBCN	Mature B-cell neoplasms	UVM	Uveal melanoma
MESO	Mesothelioma		

### Genetic alterations of FDX1

**FIGURE 2:**
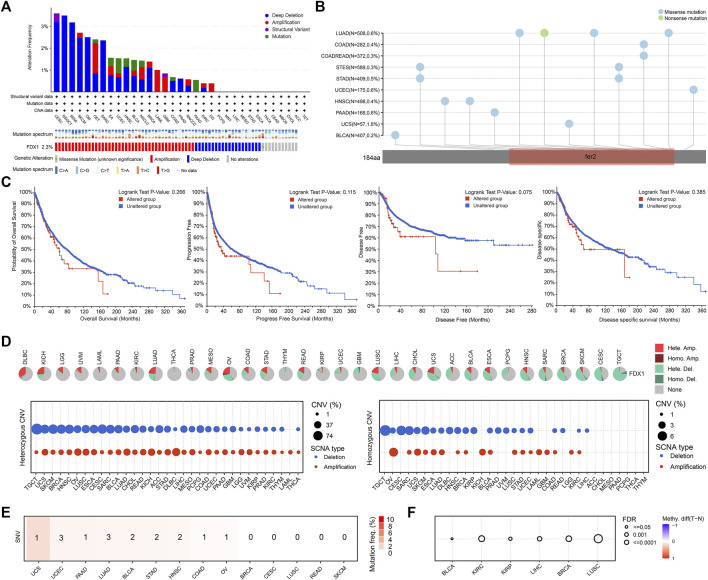
Genetic alteration of FDX1 in tumors. **(A)** The FDX1 genetic alteration frequencies of different types of tumors. **(B)** Mutation diagram of FDX1 in different tumor types across protein domains. **(C)** The survival analysis of patients with or without FDX1 genetic alterations. **(D)** Copy number variation (CNV) pie chart showed the combined heterozygous/homozygous CNV ratio of FDX1 in each tumor. The CNV profile showed the percentage of heterozygous CNV and the percentage of homozygous CNV. **(E)** The profile of single nucleotide variant (SNV) of FDX1 and number of cases with deleterious mutations in tumors **(F)** Differential methylation in FDX1 between tumor and normal samples in tumors. Red dots represented increased methylation in tumors and blue dots represented decreased methylation in tumors.

Genetic alterations are now known to be strongly associated with tumorigenesis and progression. Our analysis revealed that the average frequency of FDX1 alterations was 2.3% in all tumors, with deep deletion and amplification as the predominant types. The highest frequency of FDX1 alterations (>3%) was observed in cervical squamous cell carcinoma and endocervical adenocarcinoma (CESC), followed by nonseminomatous testicular tumor (NSGCT) and seminoma (SEMI) (Figure 2A). Further analysis showed that the main type of amino acid alteration in FDX1 was missense mutation, and most mutations affected the fer2 region. The mutation frequency was highest in LUAD, COAD and rectum adenocarcinoma (READ) (Figure 2B). However, in the pooled survival analysis of all tumor types, although the survival curves were separated, there was no statistical significance between FDX1 altered group and the unaltered group (*p* > 0.05) (Figure 2C).

To further identify FDX1 changes at the chromosomal level, we analyzed copy number variation (CNV), single nucleotide variation (SNV)and methylation level data from TCGA. As shown in Figure 2D, the major CNV types of FDX1 in 33 tumors were heterozygous amplification and deletion. In diffuse large B-cell lymphoma (DLBCL), LGG, acute myeloid leukemia (LAML), and uveal melanoma (UVM), heterozygous amplification was the main type. In pheochromocytoma and paraganglioma (PCPG), sarcoma (SARC), breast invasive carcinoma (BRCA), skin cutaneous melanoma (SKCM), CESC and testicular germ cell tumor (TGCT), the predominant type was heterozygous deletion. In addition, the proportion of homozygous FDX1 CNVs in tumors was much lower than that of heterozygous FDX1 CNVs. We also found that SNVs of FDX1 did not occur frequently in tumors, affecting no more than 4% of cases (Figure 2E). Finally, we detected differences in the methylation levels of FDX1 in only a few tumors (Figure 2F).

### RNA methylation modification-related gene analysis

**FIGURE 3:**
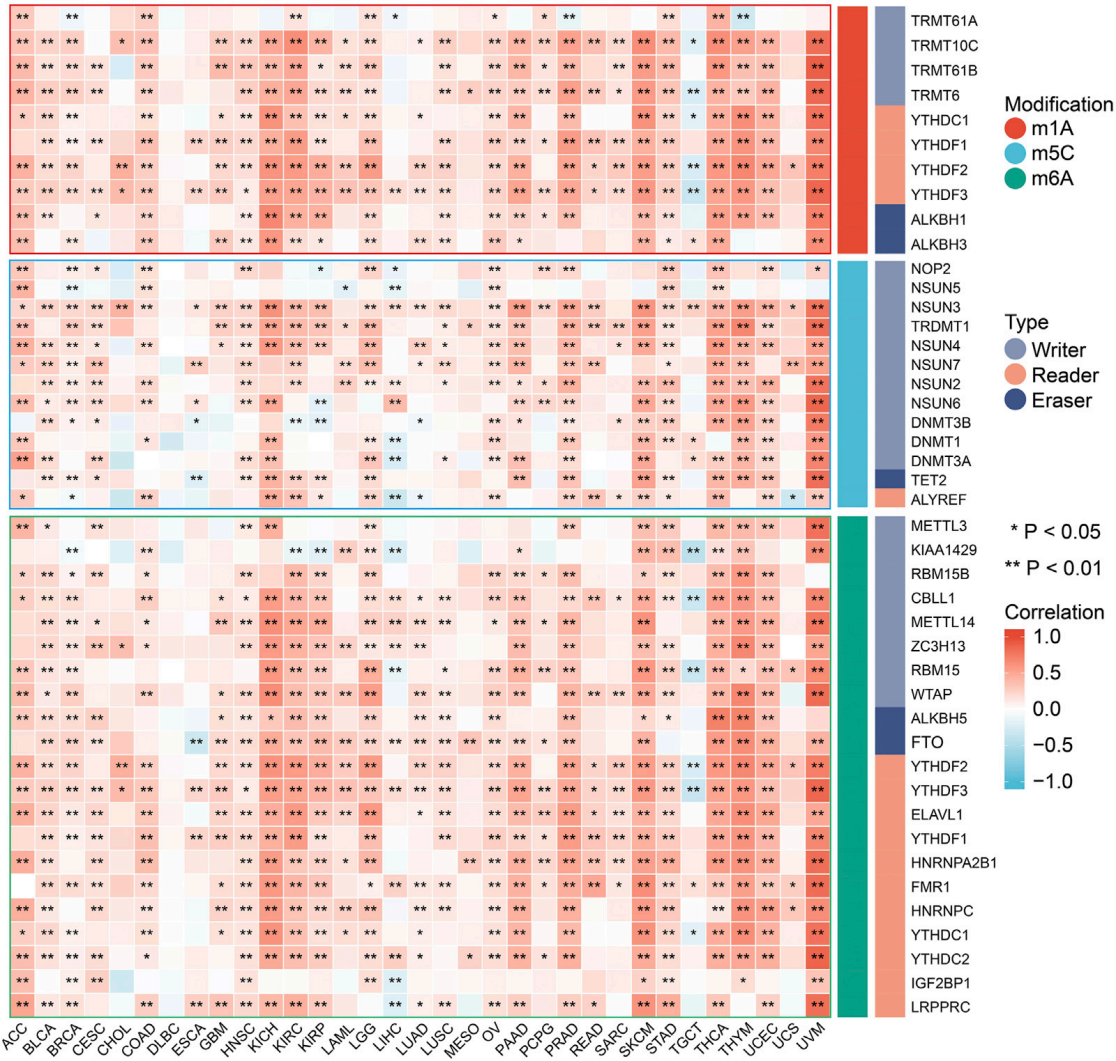
Spearman correlation of FDX1 expression with RNA methylation modifications including m1A, m5C and m6A in pan-cancer. (*p < 0.05; **p < 0.01).

Positive correlations were found between FDX1 expression and most RNA methylation modification-related genes in the vast majority of tumors (Figure 3). Only in specific tumor types, such as TGCT, did we primarily find negative correlations. In addition, FDX1 expression was not associated with any modification-related genes in DLBCL. In short, our analysis revealed that there were likely profound effects of FDX1 expression on RNA methylation modifications in tumors.

### Genomic instability analysis

**FIGURE 4:**
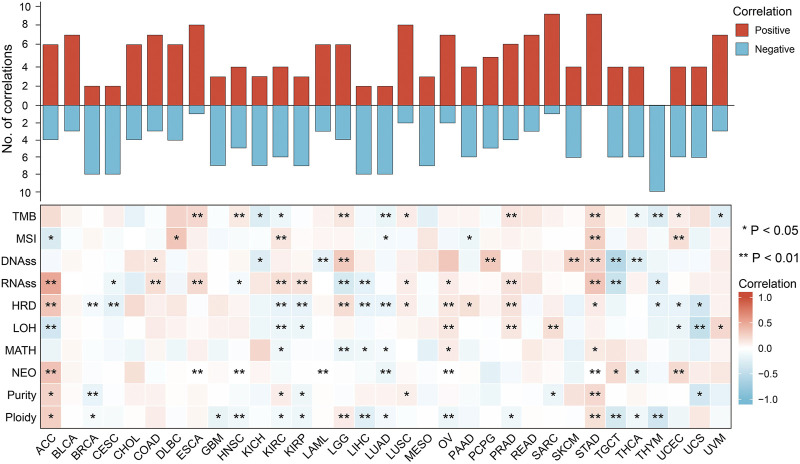
Spearman correlation of FDX1 expression with genomic instability in pan-cancer. Red indicated positive correlation, blue indicated negative correlation. And the darker the color, the stronger the correlation. (TMB, tumor mutation burden; MSI, microsatellite instability; DNAss, DNA stemness; RNAss, RNA stemness; HRD, homologous recombination deficiency; LOH, loss of heterozygosity; MATH, mutant-allele tumor heterogeneity; NEO, neoantigens; *p < 0.05; **p < 0.01).

The results of the genomic instability analysis were shown in Figure 4. The data showed significant association of FDX1 expression with genomic instability in a variety of tumors. This association was stronger in ACC, kidney renal clear cell carcinoma (KIRC) and stomach adenocarcinoma (STAD). However, in a few tumors (bladder urothelial carcinoma (BLCA), cholangiocarcinoma (CHOL), mesothelioma (MESO) and rectum adenocarcinoma (READ), FDX1 expression was not correlated with genomic instability. We found negative correlation between FDX1 expression and genomic instability parameters such as tumor mutation burden (TMB), microsatellite instability (MSI), homologous recombination deficiency (HRD), loss of heterozygosity (LOH) and neo-antigen (NEO) *etc.* in CRCA, CESC, LIHC, LUAD, TGCT, THCA and THYM. While positive correlation of FDX1 and genomic instability parameters was found in STAD, ACC, OV and PRAD. As we known, MSI and TMB are important biomarkers in immunotherapy of COAD, but there was no statistical relevance between FDX1 and TMB and MSI in COAD in our analysis.

### Associations of FDX1 expression with the levels of infiltrating immune cells in tumors

**FIGURE 5:**
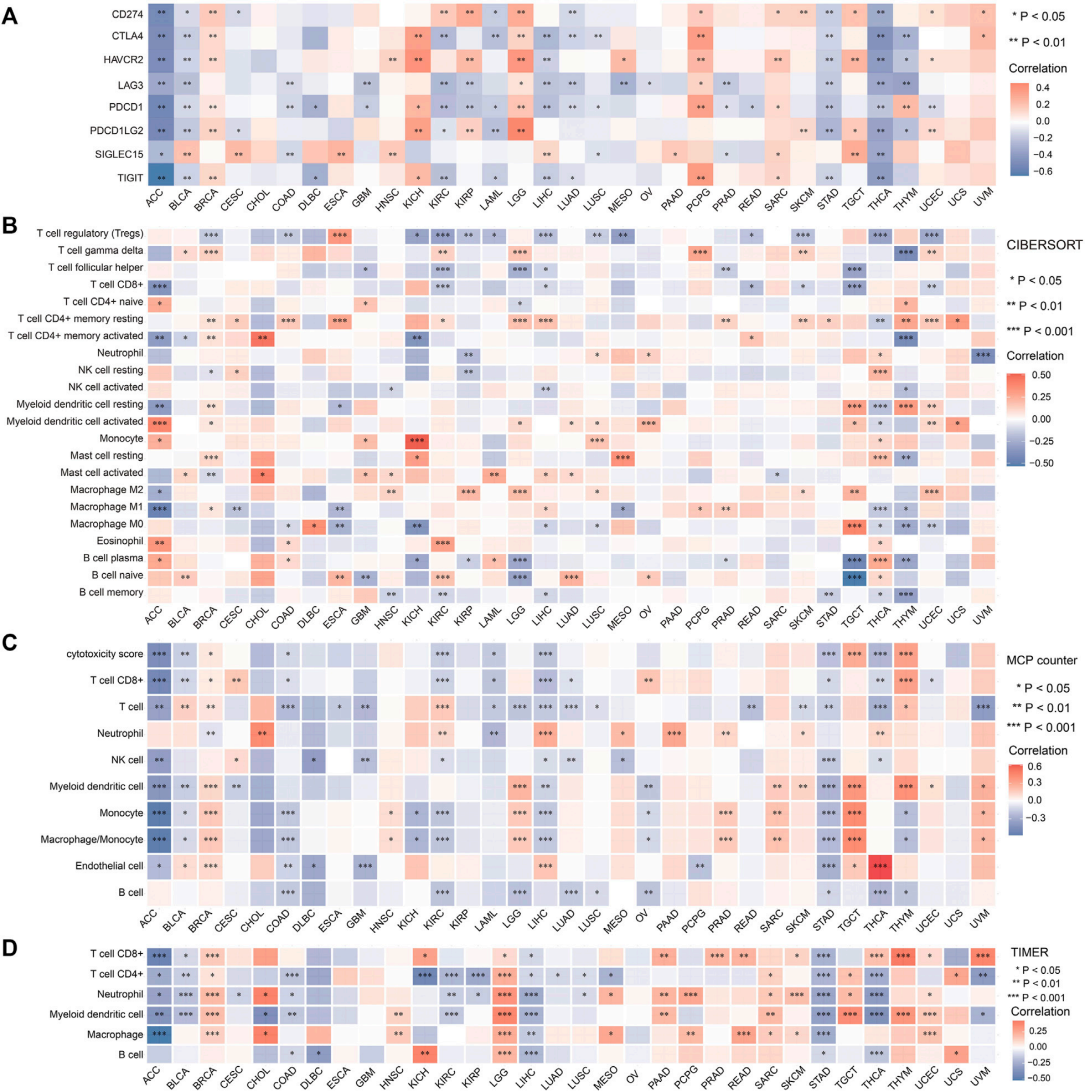
Relationship between FDX1 expression and immune checkpoints and immune cells across tumors. **(A)** Correlation of FDX1 expression and common immune checkpoint genes in pan-cancer. (*p < 0.05; **p < 0.01). Immune cell infiltration analyzed by the CIBERSORT **(B)**, MCP counter **(C)** and TIMER **(D)**. (*p < 0.05, **p < 0.01, ***p < 0.001).

FDX1 is a key molecule in cuproptosis. To determine whether cuproptosis has an effect on the immune environment of tumors, we explored the link between FDX1 expression and immune infiltration in each tumor. As shown in Figure 5A, FDX1 expression was negatively correlated with the expression of immune checkpoints such as CD274 (PD-L1), CTLA-4, TIGIT *etc.* In most tumors including ACC, BLCA, LAML, STAD and THCA *etc.*; while the expression of FDX1 was positively correlated with the expression of immune checkpoints in BRCA, KICH, LGG and PCPG *etc.*

Subsequently, we evaluated the relationship between FDX1 expression and immune cell infiltration using three algorithms. As shown in Figures 5B–D, FDX1 expression was negatively correlated with tumor infiltrating immune cells including CD8^+^ T cells, CD4^+^ T cells, macrophage *etc.* In a large part of tumor types we analyzed such as ACC, COAD, KIRC, LAML, LUAD, STAD, THCA. In most tumors, the relationship of FDX1 and immune checkpoints was consistent with its relationship with the infiltrating immune cells. However, our data showed that there were positive correlations of FDX1 and immune cells infiltration in some tumors such as LGG, SARC and TGCT etc. Also, we noted that FDX1 expression was not correlated with the expression of any immune checkpoint genes in CHOL and uterine carcinosarcoma (UCS), and the levels of infiltrating immune cells in these two tumors were also almost completely independent of FDX1 expression. These data indicating that the effect of FDX1 in tumor microenvironment varies in different type of tumors.

In the view of immune cell functions, our data showed logical results in most tumors. For example, in ACC and STAD, FDX1 expression was positively correlated with the immune effector cell CD8^+^ T cells, while it was negatively correlated with the immunosuppressive cell Tregs (Figure 5B). In ACC, COAD, LIHC and STAD, the relative consistent negative correlation of FDX1 and immune checkpoints and tumor infiltrating cells indicated the negative regulating function of FDX1 in these tumors.

### Biological process analysis

**FIGURE 6:**
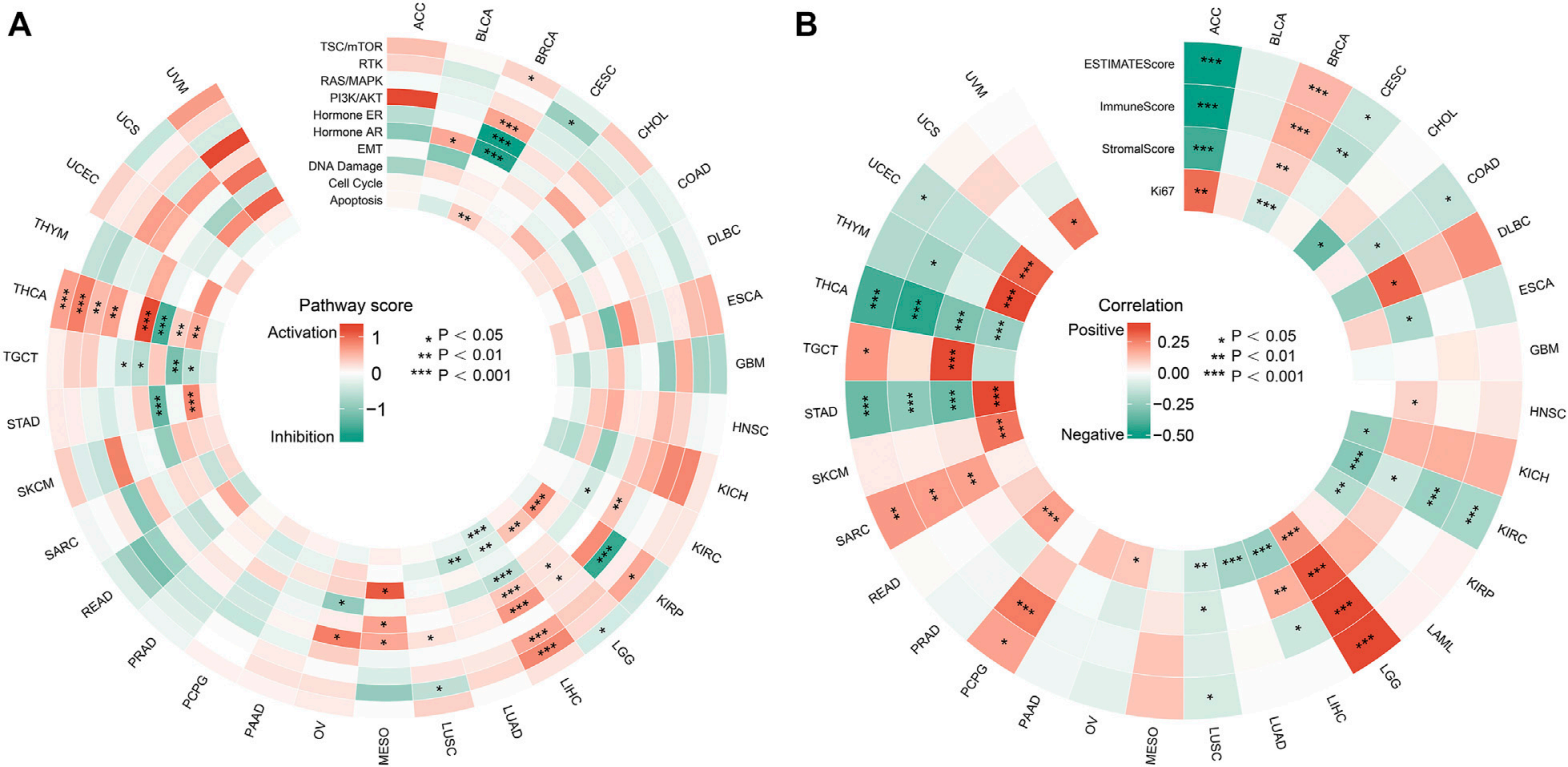
Effects of FDX1 on biological processes in tumors. **(A)** Relationship between FDX1 expression and famous cancer related pathways. (*p < 0.05; **p < 0.01; ***p < 0.001). **(B)** Correlation of FDX1 expression with tumor microenvironment scores and Ki67 in pan-cancer. (*p < 0.05; **p < 0.01; ***p < 0.001).

We first analyzed the association of FDX1 with multiply pathway activity which play important role in regulating tumor behavior in tumors. The data showed that FDX1 affected well-known tumor-associated pathways in only a small number of tumors. The effect of FDX1 was more pronounced in specific tumors, such as THCA and LIHC. In THCA and LIHC, FDX1 was positively correlated with the activity of growth promoting signaling pathways including RTK, RAS/MAPK and PI3K/AKT pathways; and was negatively correlated with DNA damage (Figure 6A). We found that FDX1 was negatively correlated with Ki-67 in LIHC and THCA (Figure 6B), and this was consisted with its role in signaling pathway. While FDX1 was positively correlated with proliferation index Ki-67 in some other tumors including ACC, LGG, PRAD, STAD, THYM and UCEC.

The tumor microenvironment (TME) has a profound impact on the biological behavior of tumors, and ESTIMATEScore, Immune Score and Stroma Score are important index to evaluate the immune state of tumors. Therefore, we investigated the correlation between FDX1 and TME parameters using the ESTIMATE algorithm. As shown in Figure 6B, FDX1 was negatively correlated with the TME scores of ACC, KIRC, THCA and STAD, while it was positively correlated with the scores of LGG and SARC. Also, the relationship of FDX1 with TME parameters and Ki-67 was in accordance in LGG, KIRC and THCA, while its role was opposite in some other tumors such as ACC, BRCA, STAD, TYHM and UCEC. All above, we can see the complexity of tumor activity and microenvironment. The specific mechanisms need to be further clarified in future studies.

### Potential functions of FDX1 in tumors

To predict the function of FDX1 in tumors, we identified genes associated with FDX1. As shown in Supplementary Figure S1A, a protein–protein interaction network based on FDX1 and ten related genes was generated by STRING. Supplementary Figure S1B shows a co-expression network consisting of FDX1 and 20 related genes. Ultimately, we obtained a total of 58 FDX1-related genes (Supplementary Figure S1C). In Supplementary Figure S1D, we present the results of functional enrichment analysis of these 58 genes and FDX1. The genes were mainly enriched in the BP terms synthesis and metabolic processes of steroids and hormones (Supplementary Figure S1E). In the CC analysis, the genes were found to be mainly located in mitochondria (Supplementary Figure S1F). The significantly enriched MF terms were iron ion binding, iron-sulfur cluster binding and metal cluster binding (Supplementary Figure S1G). As seen in Supplementary Figure S1H, the KEGG enrichment analysis revealed significant enrichment of FDX1 and FDX1-related genes in steroid hormone synthesis and secretion. The results of the functional enrichment analysis were in line with the mechanisms by which cuproptosis occurs.

In addition, we used the OPENTARGET platform to explore the diseases associated with FDX1. The results showed that in addition to its involvement in tumors, FDX1 was also involved in cardiovascular diseases, respiratory diseases, and endocrine system diseases (Supplementary Figure S1I).

### Drug sensitivity analysis

**FIGURE 7:**
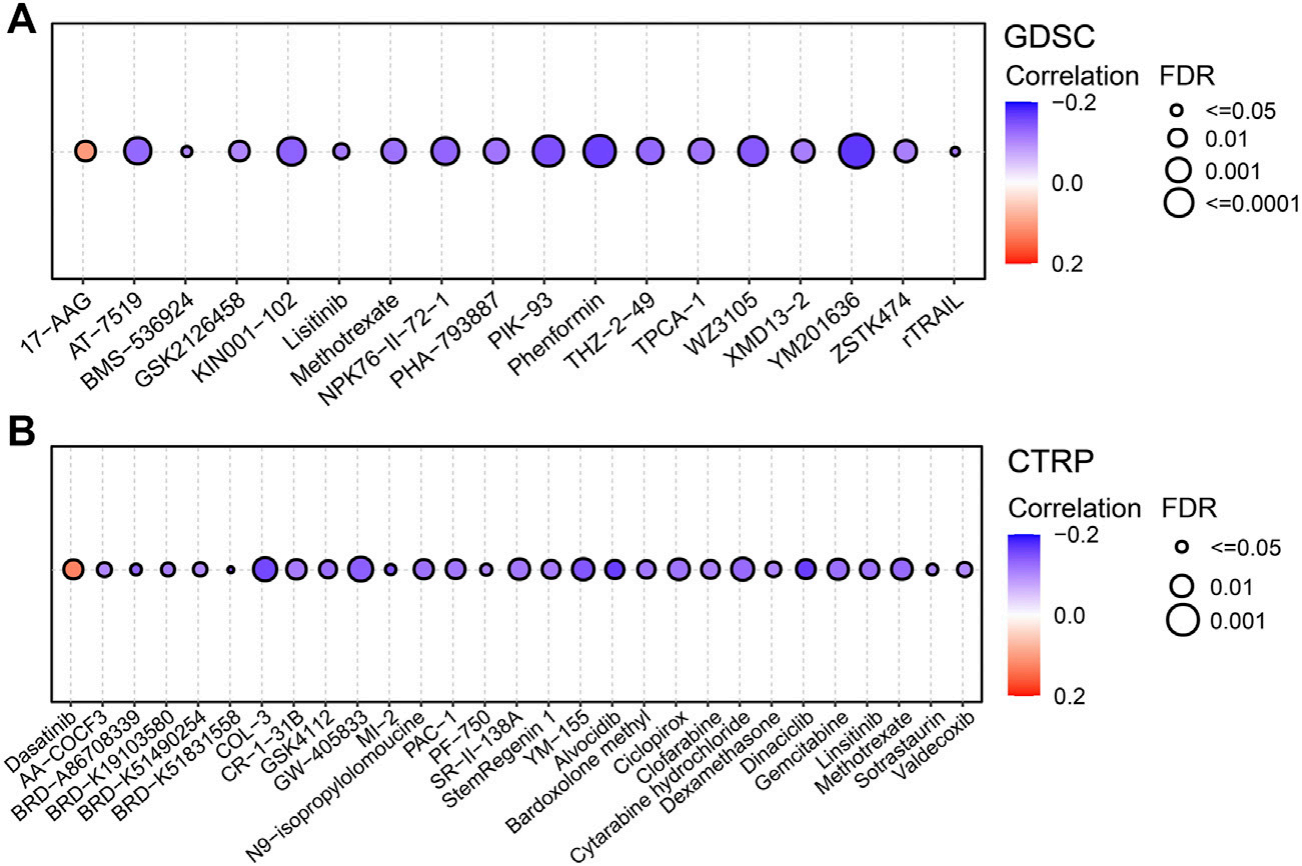
Predictive drugs based on the FDX1 expression in pan-cancer from the GDSC **(A)** and CTRP **(B)** datasets.

As shown in Figure 7A, FDX1 expression was negatively correlated with sensitivity to drugs such as AT-7519, PIK-93, phenformin and YM201636 and positively correlated with sensitivity to 17-AAG. In the CTRP database, FDX1 expression was negatively correlated with sensitivity to drugs such as ciclopirox, dinaciclib and linsitinib and positively correlated with sensitivity to dasatinib (Figure 7B). The results of TIDE analysis showed that FDX1 expression was correlated with the response to PD1 immunotherapy in a cohort of patients with melanoma and kidney tumors. More detailed data is presented in Table 2. Our analysis indicated that FDX1 had potential value for predicting chemotherapy sensitivity, but its relationship with drug sensitivity varied by cancer types.

**TABLE 2:** Association between gene expression and therapy outcome in clinical studies of immune checkpoint blockade.

Cohort	Cancer	Survival	Risk	Count	*p*
Liu2019_PD1	Melanoma	OS	2.209	47	0.027
Riaz2017_PD1	Melanoma	PFS	1.963	25	0.049
Liu2019_PD1	Melanoma	PFS	1.933	47	0.053
Miao2018_ICB	Kidney	PFS	1.892	33	0.059
VanAllen2015_CTLA4	Melanoma	OS	1.803	42	0.071
VanAllen2015_CTLA4	Melanoma	PFS	1.752	42	0.080
Gide2019_PD1+CTLA4	Melanoma	OS	1.230	32	0.219
Gide2019_PD1+CTLA4	Melanoma	PFS	1.016	32	0.310
Lauss2017_ACT	Melanoma	PFS	−1.127	25	0.260
Nathanson2017_CTLA4	Melanoma	OS	−1.336	9	0.181
Lauss2017_ACT	Melanoma	OS	−1.375	25	0.169
Nathanson2017_CTLA4	Melanoma	OS	−1.444	15	0.149
Braun2020_PD1	Kidney	OS	−2.894	295	0.038

OS, overall survival; PFS, progression free survival.

### Effects of FDX1 in tumor proliferation

**FIGURE 8:**
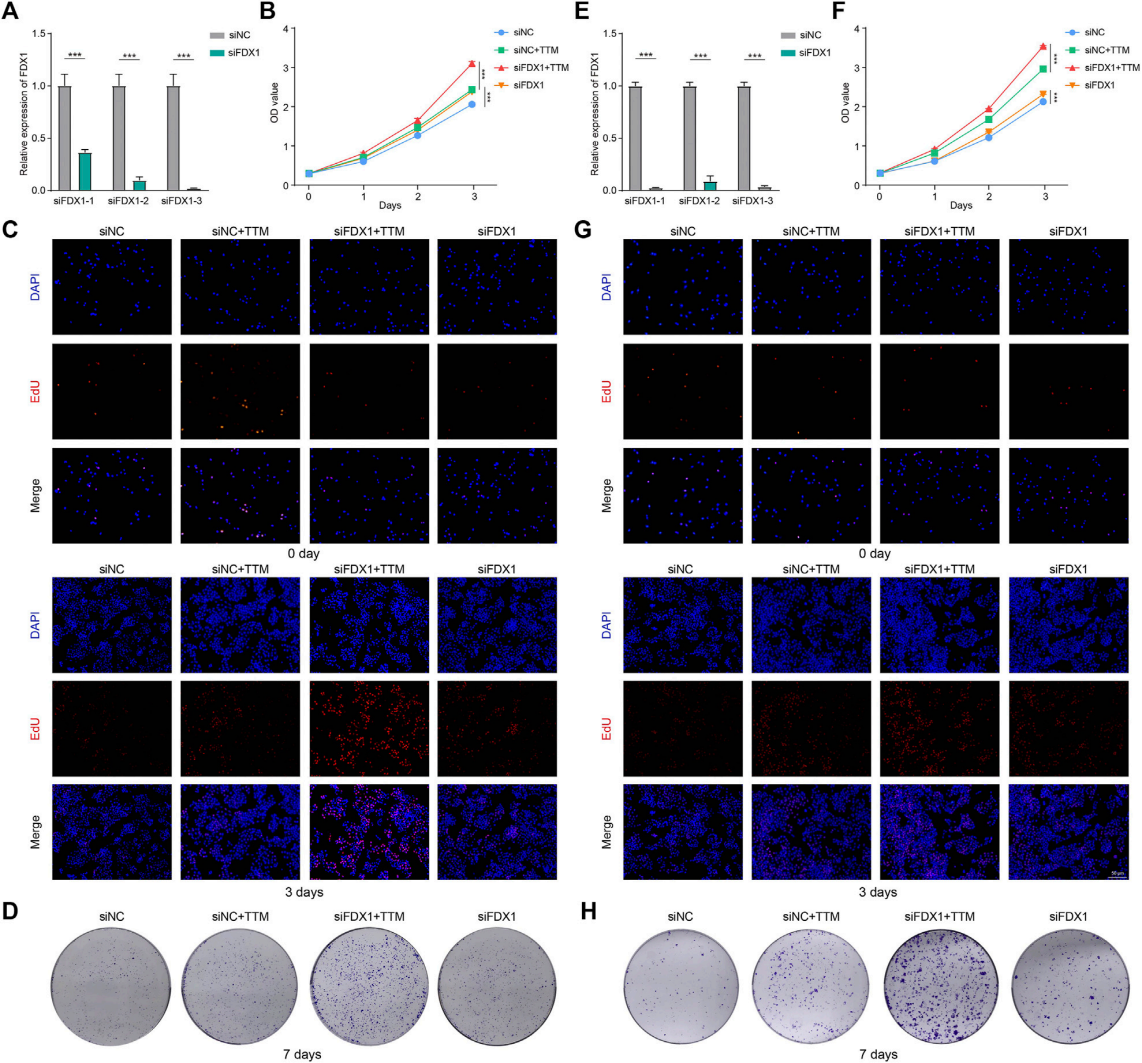
Effects of FDX1 in tumor proliferation. **(A)** PCR was performed to confirm knockdown of FDX1 in HepG2 **(A)** and H1299 **(E)** (***p < 0.001). CCK-8, EdU staining and colony formation assays were used to assess the proliferation of HepG2 **(B–D)** and H1299 **(F–H)** at specific time points (***p < 0.001).

As shown in Figures 8A,E, we successfully knocked down FDX1 in HepG2 and H1299 cell lines. And the cells with the highest transfection efficiency were selected for the subsequent experiments.

The CCK-8 showed that FDX1 inhibited the proliferation of tumor cells when copper ions were present. This inhibitory effect was significantly diminished by using TTM to chelate copper ions (Figures 8B,F). EdU staining and colony formation assays further confirmed our findings (Figures 8C,D,G,H). These results indicated that FDX1 exerted anti-tumor effects through cuproptosis.

## Discussion

FDX1 is a key molecule mediating cuproptosis. Herein, we performed the pan-cancer analysis of FDX1. First, we conducted expression analysis of FDX1 although different organs and compared the expression difference of FDX1 between normal tissues and tumor tissues. Our data showed that FDX1 expression various in different organs and tumors, indicating the heterogeneity of different organs and tumors. It is worth noticing that FDX1 protein expression was lower in COAD, GBM, HNSC, LUAD and PAAD compared with normal tissues. Together with the data in survival analysis, FDX1 expression positively correlated with survival parameters in ACC, LGG, LIHC *etc.* Also, although without statistical difference, HR was greater than one in some other tumors. We may deduce the “anti-tumor” effect of FDX1 in those tumors. This is in consistent with the study that demonstrated FDX1 was lower expressed in HCC, and FDX1 expression level was positively correlated with overall survival (Zhang et al., 2022).

In the present study, the analysis of parameters such as genomic instability and pathway activity of tumors demonstrated that FDX1 expression was closely associated with tumor biological behavior. Our data showed that the most common mutation of FDX1 was deep deletion and amplification and main type of amino acid alteration was missense mutation. Patients with genomic instability of FDX1 had the disposition to poor survival in cancer patients. There has been a research on single-nucleotide polymorphisms (SNPs) in cuproptosis-related genes demonstrating that *FDX1-rs10488764* was associated with an increased risk of lung cancer (Yun et al., 2022). Further study is needed to explore the effect of FDX1 in different tumors. Moreover, our data showed that FDX1 expression level was positively associated with cell proliferative cell pathway such as RTKs, RAS/MAPK and PI3k/AKT pathway in LIHC and THCA, while there was no significant correlation in other tumor types. Copper was reported to be a dynamic signaling metal to regulate cell behavior, for example, it was involved in mitogen-activated protein kinase 1 (MEK1) and MEK2 pathway (Smith et al., 2022). The role and mechanisms of FDX1 in cell signaling needed to explored in further studies.

Tumor microenvironment (TME) is composed of cancer cells, stromal cells, fibroblasts and innate and adaptive immune cells, producing marked effect in tumorigenesis, development and anti-tumor therapy (Hui and Chen, 2015). Previous studies have shown that cross-talk between cancer cells, stromal cells and the infiltrating immune cells made TME complex and evolving (Hinshaw and Shevde, 2019). Our study analyzed the correlation of FDX1 and tumor immune status including tumor infiltrating immune cells, immunoscore, TMB and MSI *etc*. Clinical studies have confirmed the important role of CD274 (PD-L1) expression level in immune checkpoint inhibitor (ICI) therapy in lung cancer including LUAD. Patients with higher PD-L1 expression may have better response to ICIs and tend to have better prognosis (Yang et al., 2021). In our study, PD-L1 expression was negatively correlated with FDX1 in LUAD, together with the relative lower expression of FDX1 in LUAD, inferred that PD-L1 expression might higher in FDX1 low expressed LUAD. This is an interesting project needed further validation in following researches. The effect of FDX1 to infiltrating immune cells such as CD8^+^ T cells, CD4^+^ T cells, macrophage *etc.* various in different tumors and was not exactly consistent throughout different databases. We analyze that heterogeneity may partially explain this result. On the other hand, the difference of inclusion criteria, limited sample size and lack of validation may also cause the inconsistence. And those are also the limitation of bioinformatics analysis studies.

Finally, we validated the function of FDX1 in liver hepatocellular carcinoma and non-small cell lung cancer cell lines. According to our results, knockdown of FDX1 promoted the proliferation of tumor cell lines, verifying the inhibitory effect of FDX1 in those tumors. Our result is consistent with previous studies (Tsvetkov et al., 2022; Zhang et al., 2022). It is noteworthy that Duan *et al.* declared that simply knockdown of FDX1 in LUAD neither inhibited tumor cell growth nor did it induce apoptosis (Zhang et al., 2021). Tsvetkov *et al.* reported that FDX1 knockdown partially rescued from copper induced cell-death, clarifying the important role of FDX1 in cuproptosis. We conducted our experiment with CuSO_4_, ELE and TTM, and we successfully validated the inhibitory effect of FDX1 and explored that FDX1 played its role through copper induced cell-death. In addition, there were sensitive and resistant cell lines in FDX1 mediated copper induced cell-death in Tsvetkov’s work, indicating the heterogeneity of different cell lines and the complexity of FDX1 mediated cell activity. Further studies are needed to illustrate the precise mechanism.

## Conclusion

We conducted pan-cancer analysis to explore the association between FDX1 and prognosis, genomic instability, RNA methylation modifications, immune infiltration and signaling pathway activity *etc*. Our experimental result validated the inhibitory effect of FDX1 in copper induced cell-death in liver hepatocellular carcinoma and non-small cell lung cancer cell lines. Our study reveals the functional effects of FDX1 in tumors and deepens the understanding of the effects of FDX1.

## Data Availability

The original contributions presented in the study are included in the article/Supplementary Materials, further inquiries can be directed to the corresponding author.

## Supplementary material

Supplementary Figure S1 Potential functions of RAD51 in tumors. **(A)** FDX1-interacting proteins network analysis in the STRING database. **(B)** A co-expression network of FDX1. **(C)** The union of FDX1-related genes. **(D)** Functional enrichment analysis of FDX1 and FDX1-related genes in GO and KEGG. (E–H) GO and KEGG cnetplot plot showed the top 5 enriched pathways and functions. **(I)** The OPENTARGET platform was used to conduct a gene-disease network analysis of FDX1.
